# First person – Jiang-Hua Liu

**DOI:** 10.1242/dmm.047670

**Published:** 2020-12-01

## Abstract

First Person is a series of interviews with the first authors of a selection of papers published in Disease Models & Mechanisms, helping early-career researchers promote themselves alongside their papers. Jiang-Hua Liu is first author on ‘*Akkermansia muciniphila* promotes type H vessel formation and bone fracture healing by reducing gut permeability and inflammation’, published in DMM. Jiang-Hua is a PhD student in the lab of Hui Xie in the Department of Orthopaedics, Xiangya Hospital, Central South University, China, investigating how *A*. *muciniphila* promotes type H vessel formation in callus of fractured bone.


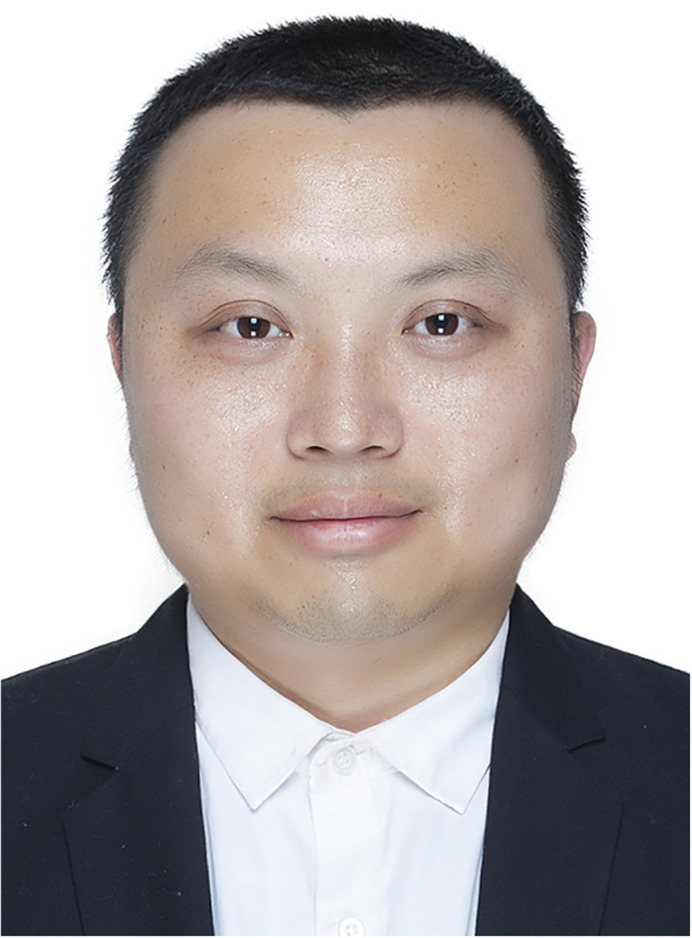


**Jiang-Hua Liu**

**How would you explain the main findings of your paper to non-scientific family and friends?**

Although the techniques and implantation materials for treating bone fractures have improved in the last few decades, a considerable number of patients still exhibit delayed healing or nonunion, which causes great medical and economic cost. New vessel formation is considered as the key factor that contributes to fracture healing, and it has been proved that better vessel formation can achieve better fracture healing. In our study, we found that *A. muciniphila*, a next-generation probiotic, can promote new vessel formation in callus, thereby improving fracture repair. We also found that the elevated new vessel formation induced by *A. muciniphila* is largely attributed to increased formation of type H vessels, a specific vessel subtype that couples vessel formation and bone formation. In addition, the pro-agiogenic and pro-osteogenic effects of *A. muciniphila* may be associated with reduced inflammation and increased preosteoclast numbers.

**What are the potential implications of these results for your field of research?**

In this study, a gut microbiome was produced that proved to be a novel regulator of the fracture healing process. Our work provides a potential effective measure to improve fracture healing, and is of certain interest and significance to researchers in tissue regeneration and applied microbiology. In addition, we were the first to demonstrate the involvement of type H vessels in fracture healing, which will probably be the new therapeutic target for improving fracture repair.

“We were the first to demonstrate the involvement of type H vessels in fracture healing, which will probably be the new therapeutic target for improving fracture repair.”

**What are the main advantages and drawbacks of the model system you have used as it relates to the disease you are investigating?**

In my opinion, using mice to establish a fracture model has two main advantages. First, the fracture healing process of mice is similar to that of humans, and internally fixing a fracture by inserting a needle is also similar to employing an intramedullary nail to treat a patient. Therefore, the model system we used in this study can appropriately simulate the process of clinical treatment for long bone fracture. Second, compared with other frequently used fracture models (such as rats, rabbits, dogs and even sheep), mice are more economical and more convenient to use for experiments.

The mouse fracture model has some disadvantages, though it has been widely used in research. For example, the needle used to fix a mouse fracture is somewhat different than that used with clinical implantation. Typically, using an intramedullary nail to treat a human fracture requires locking screws to resist rotational stress. However, the needle we used cannot resist rotational stress. Besides, mice never immobilize the injured extremity, and the stabilization of a fracture at an early stage is critical for optimal healing.

**What has surprised you the most while conducting your research?**

It was quite amazing to observe the phenomenon that *A. muciniphila* promoted fracture healing and induced more type H vessel formation, as although this capillary subtype is known to be of great importance for maintaining bone mass, no previous study has associated type H vessels with fracture repair. These data provide a new direction for fracture-related research; for example, what role do type H vessels play in fracture repair, what is the relationship between type H vessel formation and delayed union or nonunion, and do the drugs that induce type H vessel formation also promote fracture healing?

**Figure:**
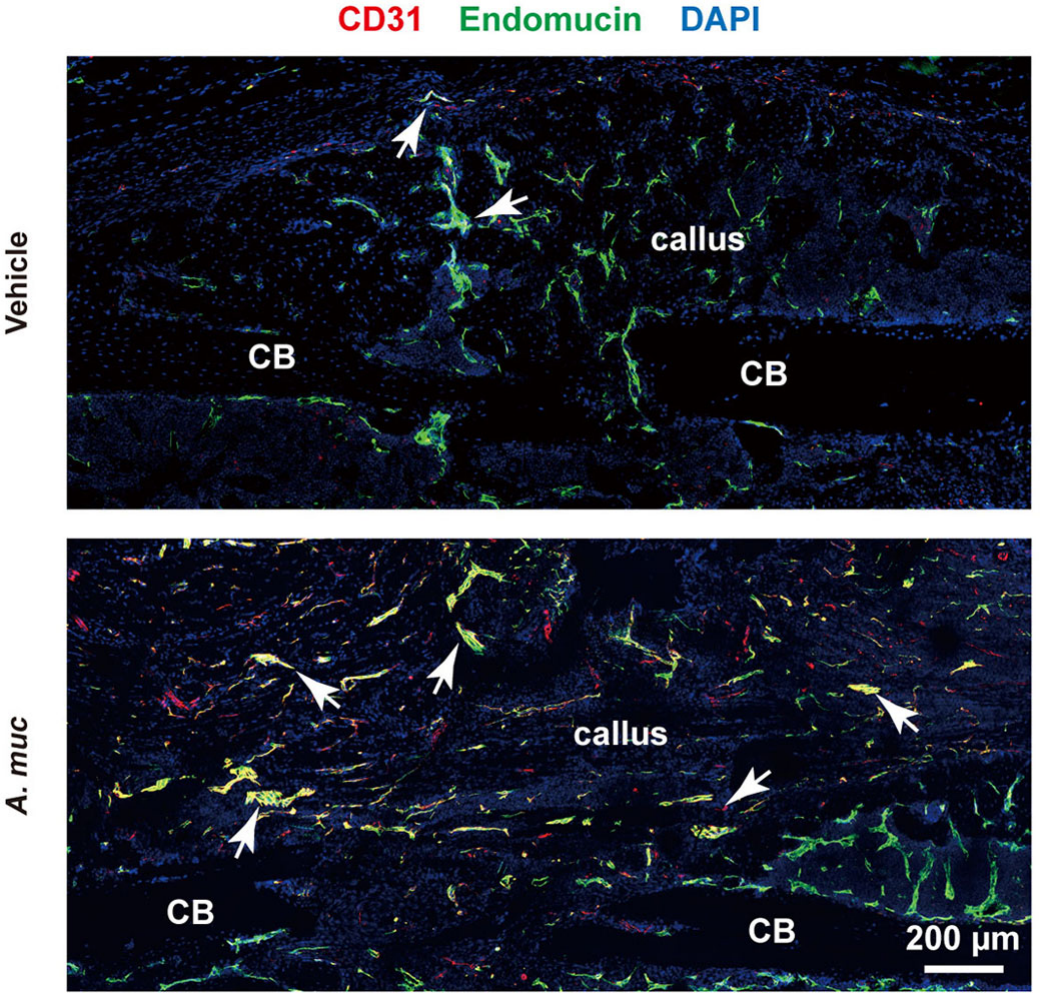
***A. muciniphila* promotes type H vessel (indicated by arrows) formation in callus of fractured bone.**

**Describe what you think is the most significant challenge impacting your research at this time and how will this be addressed over the next 10 years?**

The mechanisms of how reduced inflammation and increased preosteoclasts affect type H vessel formation remain unknown. Mice depleted of type H endothelial cells in bone, or methods to specifically inhibit this capillary subset in bone, may help researchers to address this challenge. Isolation of type H endothelial cells and culturing this subset *in vitro* will facilitate the elucidation of gene and protein expression changes of type H endothelial cells under different circumstances.

**What's next for you?**

As a former orthopaedist, once I finish my PhD, I wish to obtain a position as an orthopaedic surgeon in a research-oriented hospital, or a research position in an excellent lab, to continue my studies on probiotics and bone-related diseases. Good luck to me!
